# Immune cell phenotyping in low blood volumes for assessment of cardiovascular disease risk, development, and progression: a pilot study

**DOI:** 10.1186/s12967-020-02207-0

**Published:** 2020-01-17

**Authors:** Yvonne Baumer, Cristhian A. Gutierrez-Huerta, Ankit Saxena, Pradeep K. Dagur, Steven D. Langerman, Kosuke Tamura, Joniqua N. Ceasar, Marcus R. Andrews, Valerie Mitchell, Billy S. Collins, Quan Yu, Heather L. Teague, Martin P. Playford, Christopher K. E. Bleck, Nehal N. Mehta, J. Philip McCoy, Tiffany M. Powell-Wiley

**Affiliations:** 1 Social Determinants of Obesity and Cardiovascular Risk Laboratory, National Heart Lung and Blood Institute, National Institutes of Health, Building 10-CRC, Room 5-5332, Bethesda, MD 20892 USA; 2 Flow Cytometry Core, National Heart Lung and Blood Institute, National Institutes of Health, Bethesda, MD USA; 3 Section of Inflammation and Cardiometabolic Diseases, National Heart Lung and Blood Institute, National Institutes of Health, Bethesda, MD USA; 4 Electron Microscopy Core Facility, National Heart, Lung, and Blood Institute, National Institutes of Health, Department of Health and Human Services, Bethesda, MD USA; 5 Intramural Research Program, National Institute on Minority Health and Health Disparities, National Institutes of Health, Bethesda, MD USA

**Keywords:** Cardiovascular disease, Flow cytometry, Blood cell composition, Health disparities, Platelet adhesion

## Abstract

**Background:**

Cardiovascular disease (CVD) is the leading cause of death in the world. Given the role of immune cells in atherosclerosis development and progression, effective methods for characterizing immune cell populations are needed, particularly among populations disproportionately at risk for CVD.

**Results:**

By using a variety of antibodies combined in one staining protocol, we were able to identify granulocyte, lymphocyte, and monocyte sub-populations by CD-antigen expression from 500 µl of whole blood, enabling a more extensive comparison than what is possible with a complete blood count and differential (CBC). The flow cytometry panel was established and tested in a total of 29 healthy men and women. As a proof of principle, these 29 samples were split by their race/ethnicity: African-Americans (AA) (N = 14) and Caucasians (N = 15). We found in accordance with the literature that AA had fewer granulocytes and more lymphocytes when compared to Caucasians, though the proportion of total monocytes was similar in both groups. Several new differences between AA and Caucasians were noted that had not been previously described. For example, AA had a greater proportion of platelet adhesion on non-classical monocytes when compared to Caucasians, a cell-to-cell interaction described as crucially important in CVD. We also examined our flow panel in a clinical population of AA women with known CVD risk factors (N = 20). Several of the flow cytometry parameters that cannot be measured with the CBC displayed correlations with clinical CVD risk markers. For instance, Framingham Risk Score (FRS) calculated for each participant correlated with immune cell platelet aggregates (PA) (e.g. T cell PA β = 0.59, p = 0.03 or non-classical monocyte PA β = 0.54, p = 0.02) after adjustment for body mass index (BMI).

**Conclusion:**

A flow cytometry panel identified differences in granulocytes, monocytes, and lymphocytes between AA and Caucasians which may contribute to increased CVD risk in AA. Moreover, this flow panel identifies immune cell sub-populations and platelet aggregates associated with CVD risk. This flow cytometry panel may serve as an effective method for phenotyping immune cell populations involved in the development and progression of CVD.

## Background

Cardiovascular disease (CVD) is the leading cause of death worldwide. An estimated 17.3 million people die from CVD each year, accounting for 31.5% of all deaths [1]. In the United States, CVD disproportionately affects African Americans [2]. Despite significant advances in the prevention and treatment of CVD, racial/ethnic disparities in both the incidence of and mortality from CVD still persist [2]. Data shows that African Americans live 3.4 years less than Caucasians, with a significant proportion of the mortality difference attributed to CVD. Specifically, African Americans have a higher prevalence of hypertension, obesity, and diabetes which contributes to deleterious health outcomes, including stroke, chronic kidney disease, and congestive heart failure [3, 4]. African Americans are also overburdened by chronic psychosocial and environmental stressors that influence CVD outcomes independent of CVD risk factors or unhealthy behaviors [5]. Immune responses to adverse psychosocial and environmental conditions likely contribute to the increased risk of CVD among African Americans [6].

In recent years, researchers have made tremendous advances in understanding the role of inflammation and the immune system in CVD [7, 8]. Almost all cells of the immune system have been linked to the development of atherosclerosis [7, 9–18]. More recent literature also demonstrates how changes in clonal hematopoiesis may be a potential risk factor for CVD [19–22], especially in an aging population. Additionally, changes in clonal hematopoiesis likely alter blood cell distribution and function. Given the importance of immune cells in CVD development, minimally invasive assays using low blood volumes are critically needed to measure changes in blood cell distribution, immune function and, ultimately, CVD risk in response to atherosclerosis therapy [23].

Thus, we have developed a minimally-invasive flow cytometry panel which requires a low volume of blood and aids in phenotyping immunologic and hematopoietic cell populations involved in CVD development and progression [7, 10–12, 16, 24–29]. This assay provides a significant amount of information using 500 µl of blood. As an example of the utility of this technique, we first demonstrate hematologic differences at baseline between African American and Caucasian blood donors that we hypothesize may contribute to increased CVD risk in African Americans. Furthermore, we examine the associations between parameters identified using the flow cytometry panel as compared to a traditional complete blood count with differential (CBC) and markers of CVD risk [i.e. Framingham Risk Score (FRS) and high-sensitivity C-reactive protein (hsCRP)] in a population of African American women at increased risk for CVD.

## Methods

### Human subjects

Study approval was obtained from the Institutional Review Board (IRB) at National Heart, Lung and Blood Institute (NHLBI), National Institutes of Health (NIH) in accordance with the principles of the Declaration of Helsinki. All guidelines for good clinical practice and in the Belmont Report (National Commission for the Protection of Human Subjects of Biomedical and Behavioral Research) were followed. Data for all study participants were obtained under the IRB approved clinical trials NCT01143454, NCT03288207, and NCT00001846. All study participants in the cohort provided written informed consent. Blood bank donors were de-identified prior to distribution.

### Establishing flow panel

#### Antibody titration

For antibody titration, cells were isolated from whole blood and stained with each antibody of the flow panel in a two-fold serial dilution and analyzed as described in Additional file 1: Figure S1. 20 ml of blood was added to 4 × 50 ml Falcon tubes and in each tube 45 ml of ACK (10-548E, BioWhittaker, USA) lysis buffer was added and incubated for 2 min at room temperature (RT). Red blood cell lysis was repeated for 2 min at RT with subsequent centrifugation at 300×*g* for 4 min at RT. Cells were resuspended in 1 ml of flow buffer each (flow buffer 1L: PBS pH7.4 with 500 µl 0.5 M EDTA pH8.0 and 0.2% BSA). Live isolated cells were counted using a hemocytometer (CP2-002, Cellometer, Nexcelom, USA) after Trypan blue (25-900-02, Corning, USA) staining. Subsequently, isolated cells were diluted to 0.2 × 10^6^ cells/100 µl in flow buffer, with the antibody dilutions prepared as described in Additional file 1: Figure S1A, and 100 µl of cell suspension added to each well of the 96-well round bottom plate. Antibodies and cells were incubated for 20 min at 4°C in the dark. Afterwards, cells were centrifuged at 300×*g* for 4 min at RT, the supernatant discarded, and washed using 200 µl flow buffer. After a final centrifugation wash step, cells were resuspended in 200 µl flow buffer containing 1% paraformaldehyde (PFA) fixative in flow buffer (D2650, Sigma Aldrich, USA). Flow cytometry was performed using the LSR Fortessa (BD Bioscience, USA) and resulting analysis histograms are displayed in Additional file 1: Figure S1B.

#### Compensation

Multi-color flow cytometry and use of several fluorochrome tagged antibodies will require the setup of a compensation panel to account for fluorochrome emission spillover from one channel into the other. For compensation purposes, One Comp E beads (101-1111-42, Invitrogen, USA) were used. One drop of beads was added to each individually labeled flow tube (3520588, Falcon Corning, USA) and the included antibodies (amounts from Table 1) were added to a tube containing the Comp E beads and incubated for 15 min at RT in the dark. In order to prepare a positive control for the yellow live/dead staining (L34968, Invitrogen, USA), 1 × 10^6^ cells isolated from whole blood were incubated with 20% DMSO (D2650, Sigma Aldrich, USA) for 15 min at RT, and afterwards stained for live/dead (3.5 µl in 1 ml flow buffer) for 15 min at RT in the dark. Labeled compensation beads, stained cells, and an unstained sample of cells were analyzed using the LSR Fortessa (BD Bioscience, USA) compensation mode.

**Table 1:** Summary of antibodies/fluorochromes used in this study

Target cell type	Antigen	Fluorochrome	clone	Supplier (Cat No)	Amount (µl)	Isotype control
T cell	CD3	PECy7	UCHT1	EBioscience (25-0038-42)	5	Mouse IgG1 k-PECy7
Monocyte	CD14	APC	61D3	EBioscience (17-0149-42)	5	Mouse IgG1 k-APC
Neutrophil	CD15	BV786	HI98	BD Biosciences (563838)	2.5	Mouse IgM-BV786
Monocyte/NK cell, Neutrophil	CD16	BUV395	3G8	BD Biosciences (563785)	2.5	Mouse IgG1 k-BUV395
B cell	CD19	BV650	SJ25C1	BD Biosciences (563226)	5	Mouse IgG1 k-BV650
Platelet	CD42b	BV421	HIP1	BioLegend (303930)	2.5	Mouse IgG1 k-BV421
Leukocyte	CD45	PerCP-cy5.5	2D1	BD Biosciences (340953)	30	Mouse IgG_1_, k-PerCP-cy5.5
NK cell	CD56	FITC	NCAM16.2	BD Biosciences (340410)	10	Mouse IgG2b-FITC
Eosinophil	CD193	APCCy7	5E8	BioLegend (310712)	5	Mouse IgG2b-APCCy7
Basophil	CD203c	PE	NP4D6	BioLegend (324606)	10	Mouse IgG1 k-PE
					Total: 77.5	

#### Fixation of samples

The fixation of samples before running the flow cytometry analysis is in most laboratories common practice as often samples have to be prepared in advance. Therefore, we performed a setup test comparing fixed and non-fixed samples side-by-side (Additional file 1: Figure S2). Cells from 2 ml whole heparinized blood were isolated and stained with each antibody in duplicate for 20 min at 4 °C in the dark. In the final step, samples were resuspended in 200 µl flow buffer containing 1% PFA fixative, while sample II was resuspended in 200 µl flow buffer without fixative added. Flow cytometry was performed using the LSR Fortessa (BD Bioscience, USA) immediately on non-fixed samples, while fixed samples were held for 24 h at 4 °C in the dark.

#### Isotype backbone verification

**Fig. 1:**
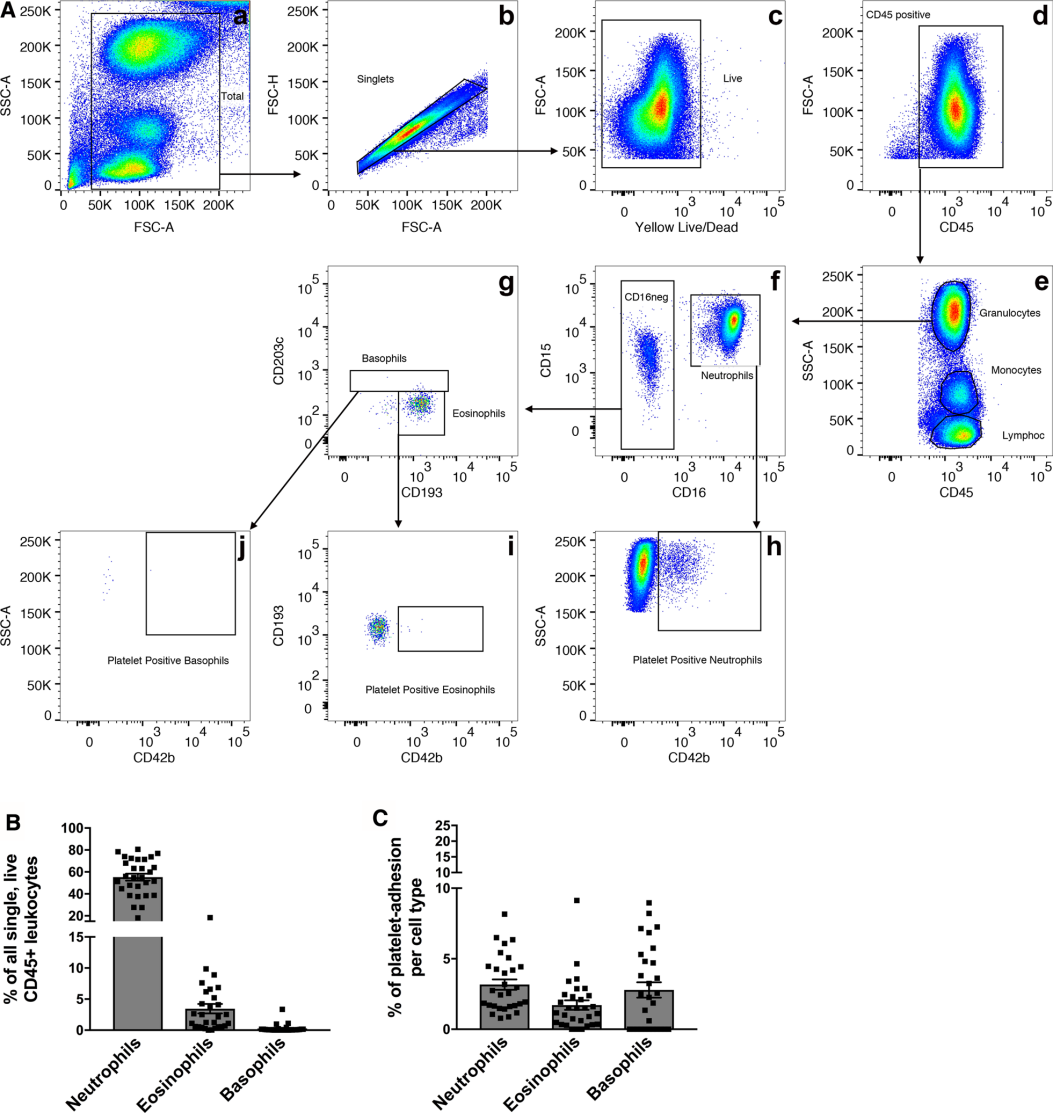
Granulocyte phenotyping. **A** Representative example of flow cytometry gating scheme to identify granulocytes. Each cell subsets CD42b (platelet)-positive population can also be identified. **B** Identified cell populations are presented in percent (%) of all CD45-positive cells displayed in **A**, **d**. **C** Platelets adherent to each cell population are presented in percent (%) of originating gate **A**, **f**, **g**. Representative quantitative results of 29 healthy adult blood donors. Data are represented as mean ± the standard error of the mean

**Fig. 2:**
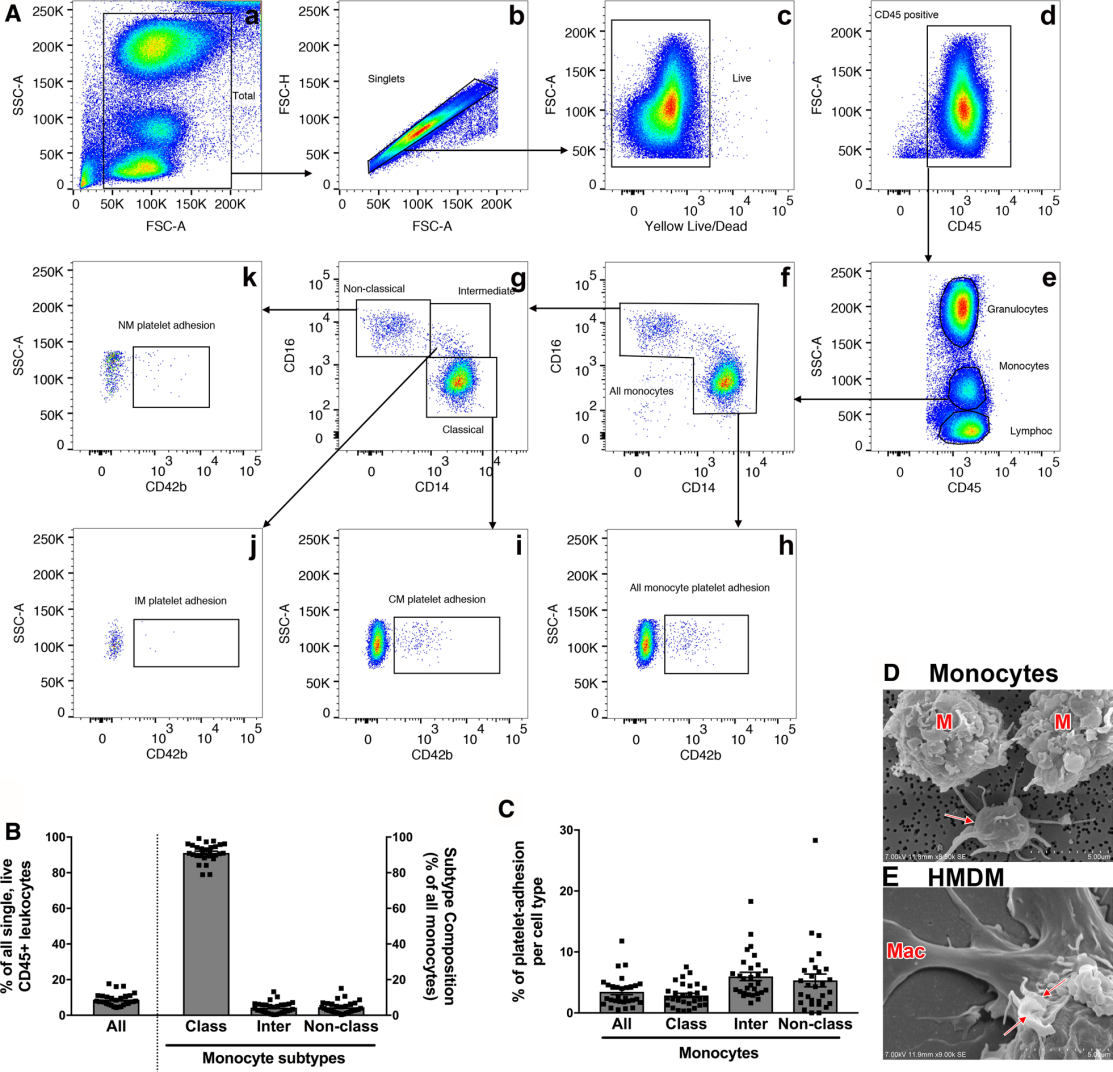
Monocyte phenotyping. **A** Representative example of flow cytometry gating scheme to identify monocytes and their subsets (**A**, **f**, **g**). Each cell subsets CD42b (platelet)-positive population can also be identified (**A**, **h**–**k**). (**B**) Identified cell populations are presented in percent (%) of all CD45-positive cells displayed in (**A**, **d**). **C** Platelets adherent to each cell population are presented in percent (%) of originating gate (**A**, **f**, **g**). **D**, **E** Scanning electron micrographs displaying the monocytes with adherent platelets. Adherent platelets are indicated by the red arrows and stay adherent on monocytes during the process of macrophage differentiation (**E**). Representative quantitative results of 29 healthy adult blood donors. Data are represented as mean ± the standard error of the mean. (NM-nonclassical monocytes, IM-intermediate monocytes, CM-classical monocytes HMDM-human monocyte-derived macrophages)

**Fig. 3:**
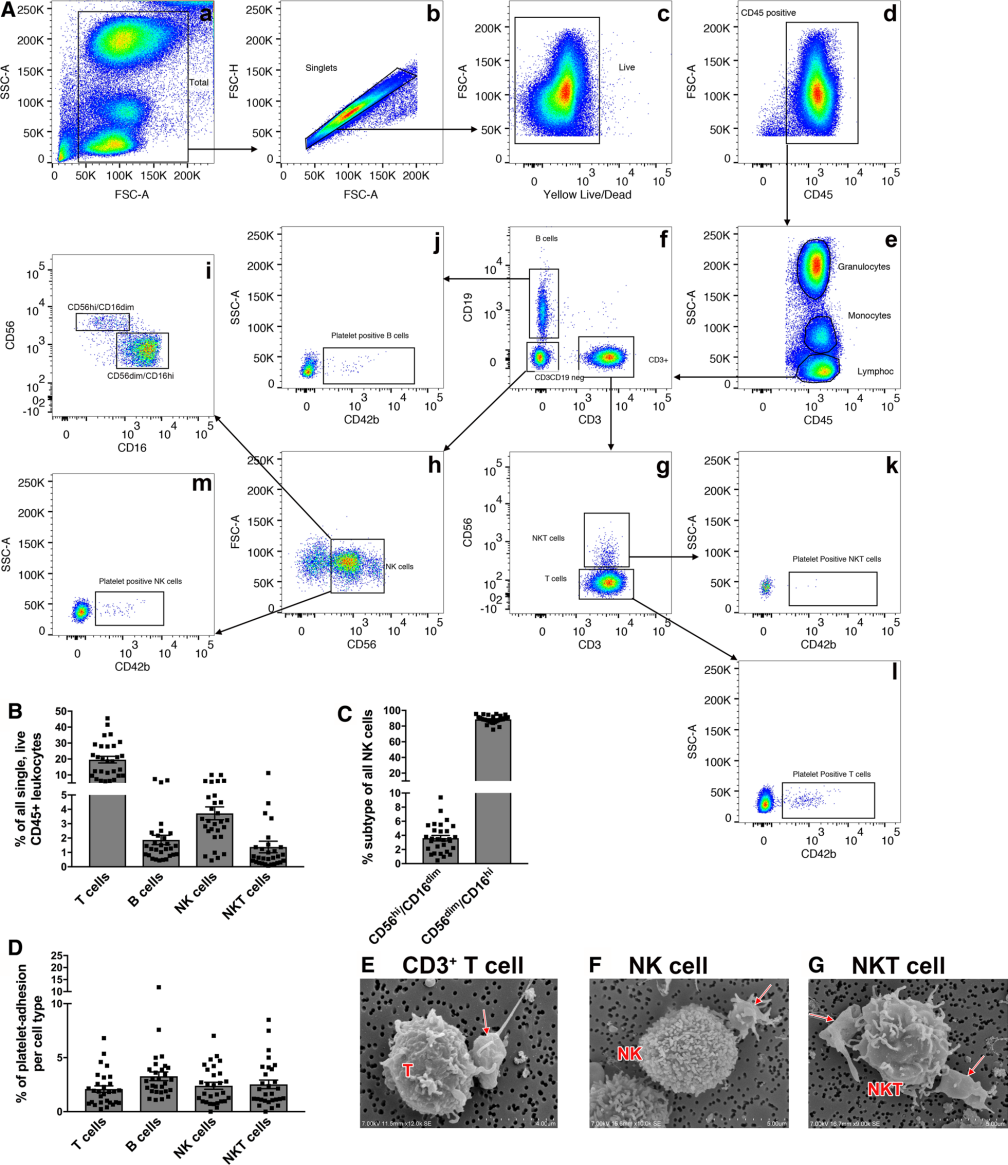
Lymphocyte phenotyping. **A** Representative example of flow cytometry gating scheme to identify B cells (**A**, **f**), T cells (**A**, **g**), NKT cells (**A**, **g**), and NK cells (**A**, **h**). NK cells can further be sub-gated to allow identification of cytotoxic (CD56dim/CD16high) or proliferative NK cells (CD56high/CD16dim) (**A**, **i**). Each cell subsets CD42b (platelet)-positive population can also be identified (**A**, **j**–**m**). **B** Identified cell populations are presented in percent (%) of all CD45-positive cells displayed in (**A**, **d**). **C** Sub-gating of NK cells (**A**, **h**) by CD56 and CD16 allows for quantification of proliferative versus cytotoxic NK cell populations displayed as percent of CD3-/CD56+ NK cells (**A**, **h**). **D** Platelets adherent to each cell population are presented in percent (%) of originating gate. Representative quantitative results of 29 healthy adult blood donors. **E**–**G** Scanning electron micrographs displaying the indicated population and the adherent platelet. Adherent platelets are indicated by the red arrow. Data are represented as mean ± the standard error of the mean

We used a technique named fluorescence-minus-one to ensure that obtained staining results were indeed due to antigen-antibody binding and not due to nonspecific binding of the antibody’s backbone or Fc receptors (isotype controls). Staining was done for 20 min at 4 °C in the dark. Flow cytometry was performed using the LSR Fortessa (BD Bioscience, USA). The gating scheme illustrated in Figs. 1, 2 and 3 was then applied and used to analyze all samples. Isotype-stained samples were contrasted with full panel-stained samples to identify the possible presence of nonspecific binding. Histograms of the correspondent parent gate were then obtained to verify positive selection per antibody and are displayed in Additional file 1: Figure S3.

### Sample collection from whole blood for flow cytometry

For study participants, we aim to characterize immune cells distribution in the blood. We therefore drew blood into ‘Green Top’ blood collection tubes and processed those as described below for flow cytometry. Blood was used within 2 h from drawing for immunophenotyping. 0.5 ml of whole blood was transferred into a 50 ml conical falcon tube for flow cytometry, 10 × the volume of blood ACK lysis buffer (10-548E, BioWhittaker, USA) added, and incubated for 2 min at room temperature (RT). Afterwards the samples were centrifuged at 300×*g* for 4 min at RT, the supernatant discarded, new ACK lysis buffer added and incubated for 3 min at RT. After another centrifugation step at 300×*g* for 4 min at RT the supernatant was discarded. The pellet was washed using 10 ml flow buffer (1L: PBS pH7.4 with 500 µl 0.5 M EDTA pH8.0 and 0.2% BSA) with subsequent centrifugation for 4 min at 300×*g* at RT. The antibody cocktail was prepared in 200 µl of flow buffer using the appropriate antibody volumes as listed in Table 1. Cells were then transferred into a flow tube for fluorescent staining. The staining process was completed at 4 °C for 20 min. The cells were then washed in flow buffer and resuspended in 1% PFA fixative in flow buffer. Flow cytometry was performed using the LSR Fortessa (BD Bioscience, USA).

### Data compilation using FlowJo™10 software

All flow cytometry data analysis was performed using FlowJoTM10 software (FlowJo LLC, USA). The correct parent gate was identified as the gate encapsulating all leukocytes so that appropriate proportional comparison may be assessed. In particular, the gate that encapsulates all non-debris, single, live, CD45-positive cells was termed the parent gate. The cell count for this parent gate was then obtained and used as a reference point for total cells acquired per sample. Using CD-marker expression and the gating scheme displayed in Figs. 1, 2 and 3, cell count per leukocyte subset was obtained. The leukocyte subset cell count was then divided by the total cells acquired to obtain proportional references per subset per sample. Results are represented as percentages of all non-debris, single, live, CD45-positive cells.

### Scanning Electron Microscopy (SEM)

To visualize some of our cell populations from flow cytometry data, we opted to perform scanning electron microscopy (SEM). CD3-positive T-cells were isolated from heparinized whole blood using REAlease CD3 Microbead kit (130-117-038, Miltenyi, USA) with subsequent CD56 bead isolation to separate CD3-positive T-cells from CD3/CD56-positive NKT-cells per manufacturers recommendation. Natural killer (NK) cells were isolated using CD56 beads (130-050-401, Miltenyi, USA) following the manufacturers recommendations from the flow-through derived after CD3 positive cell selection. Monocytes were isolated from CD3/CD56 negative flow through derived after all isolation steps using the Easy Sep™ Monocyte enrichment kit (19058, StemCell technologies, USA). Each isolated cell type was allowed to recover from the isolation steps overnight at 37 °C/5% CO_2_ in X-Vivo-15 media (Lonza, USA). In another set of experiments PBMCs were differentiated into macrophages based on the adhesion method as described previously and cultivated for 7 days in vitro in X-Vivo15 media supplemented with 20% FBS [30].

For each setup, cells were fixed using 2.5% glutaraldehyde (0.1 M calcium chloride, 0.1 M sodium cacodylate buffer, pH 7.2) for 1 h at RT and prepared as described previously [30–33] using polypropylene syringe filter holders (Cat: Pall Life science 4317, 13 mm Swinney, USA) with an inserted 0.1 μm disk filter (Pall Life Sciences, USA) and 1 ml insulin syringes (SS1D2516, Terumo, USA). Then samples were coated with 5 nm gold/palladium using the EMS 575-X sputter coater (Electron Microscopy Sciences, Hatfield, USA). Cells were imaged using the Hitachi S-3400N1 SEM at 7.5 kV (NHLBI Electron Microscopy Core).

### Clinical assessments

Data regarding the demographics, clinical histories, and anthropometric measurements of study participants were collected per clinical protocols NCT01143454, NCT03288207, and NCT00001846 at the NIH Clinical Center. Laboratory parameters used in this study (hsCRP, CBC, etc.) were measured in the NIH Clinical Center Department of Laboratory Medicine. Framingham risk score (FRS) was used to investigate the participant’s 10-year risk for cardiovascular disease. FRS were calculated as previously described using six coronary risk factors including age, gender, total cholesterol (TC), HDL-cholesterol, systolic blood pressure, and smoking habits [34].

### Statistical analysis

Statistical comparison between groups was performed using PRISM 7.0 (GraphPad) software. Data are represented as mean ± the standard error of the mean. Statistical significance was evaluated using Mann-Whitney statistical test. Statistical significance was established at a p value of p < 0.05 (*p < 0.05, **p < 0.005, ***p < 0.0005). Exact n-numbers are given in the Figure legends. The obtained flow cytometry data were analyzed by two independent researchers in a blinded fashion. All statistical correlations were performed blinded. Unadjusted and adjusted multivariable linear regression models were performed on STATA release 12 (StataCorp., College Station, Tx, USA). Multivariable linear regression analyses were used to evaluate the associations of CBC and flow cytometry derived measures with either FRS or hsCRP. P values of ≤ 0.05 are reported as statistically significant.

## Results

### Initial steps of establishing a flow cytometry panel

As a first setup step, it was important to determine the optimal concentration of antibody needed for staining a predetermined number of cells. False positives or false negatives could have occurred if incorrect antibody amounts were used; verifying antibody amounts was also necessary as optimal antibody concentrations could have been below the manufacturer’s recommended concentration. In order to assess which concentration yielded optimal results, a serial dilution plate was prepared as described in Additional file 1: Figure S1A. The optimal concentration was determined to be the antibody concentration which produced the highest positive signal without increasing the background (Additional file 1: Figure S1B). Optimal concentrations (summarized in Table 1) were then used on a sample for compensation configuration, and ultimately, for blood sample analyses. Next, it was important to check if stained samples could be fixed and analyzed the following day (Additional file 1: Figure S2). We determined that fixation did not affect the quality of the data. Additionally, we had to ensure that staining results were indeed due to antigen-antibody binding and not due to nonspecific binding of the antibody’s backbone or Fc receptors (Additional file 1: Figure S3). We found that cells stained with isotype controls displayed a clear separation and shift to the right compared to the fully stained sample and parent gate, indicating the effectiveness of the chosen antibodies and clones. Illustrative histograms are displayed in Additional file 1: Figure S3. As to the stability of the identified immune cell distribution during storage or transport of donor blood, the literature suggests that a maximum of 72 h storage at RT might be possible [35]. However, to ensure optimal validity of this flow panel, our data suggest sample processing needs to be performed within 5 h of drawing the blood (Additional file 1: Figure S4).

### Flow cytometry analysis: Granulocytes

First, we focused on the analysis of granulocytes following the gating scheme demonstrated in Fig. 1. Among 29 blood donors, we found that granulocytes comprised the largest group of all cells. Neutrophils made up the largest leukocyte population accounting for 55.23 ± 3.12% of all single, live, CD45-positive cells. Eosinophils comprised 3.45 ± 0.76% and basophils 0.26 ± 0.12% of all single, live, CD45-positive cells. The addition of CD42b in our flow cytometry panel also allowed us to identify the presence of platelet-adherence on all presented cell populations (Fig. 1A, h–j). Taking the data from the 29 blood donors, we identified that an average 3.17% ± 0.37 of neutrophils, 1.71% ± 0.34 of eosinophils, and 2.78% ± 0.56 of basophils had platelets adherent to their surface.

### Flow cytometry analysis: monocytes

The second step of the flow cytometry analysis focused on monocytes from debris-free, single, live CD45-positive cells as demonstrated in Fig. 2A, a–e. Monocytes can be further identified and subdivided by their CD16 and CD14 expression (Fig. 2A, f). Non-classical monocytes are characterized by a high CD16 expression and low CD14 expression while intermediate monocytes are characterized by high CD14/CD16 expression (Fig. 2A, g). Analysis of 29 blood donors (Fig. 2B), shows that monocytes comprise 8.74 ± 0.65% of all single, live, CD45-positive cells. Of all monocytes, 91.12 ± 0.93% are classical monocytes followed by non-classical monocytes, 4.52 ± 0.63%, and intermediate monocytes, 4.32 ± 0.58%.

CD42b expression was further employed to assess monocyte-platelet aggregates (Fig. 2A, h–k, C). This analysis showed that platelet adherence on all monocytes was 3.46 ± 0.46%, on classical monocytes was 2.86 ± 0.36%, on intermediate monocytes was 5.98 ± 0.68%, and on non-classical monocytes was 5.33 ± 1.05%. This phenomenon was confirmed and visualized via scanning electron microscopy (SEM) (Fig. 2D, E). When imaging monocytes by SEM, platelets attached or adherent to monocytes can be visualized. Often several platelets are adherent to one single monocyte. Interestingly, platelets adherent to monocytes do not detach even after 6 days of differentiation into human monocyte-derived macrophages and can therefore still be visually identified after complete macrophage differentiation (HMDM, Fig. 2E).

### Flow cytometry analysis: lymphocytes

Finally, characterization and phenotyping of our samples’ lymphocytes was done by gating cells based on SSC-A and CD45 expression after excluding debris, duplets, and dead cells following the gating scheme shown in Fig. 3A. Calculating the cell proportions of each cell type in reference to our parent gate, our 29 blood donor samples consistently showed that a large portion of lymphocytes were T cells, averaging 19.61 ± 2.08%, followed in frequency by natural killer (NK) cells, B cells, and NKT cells averaging 3.72 ± 0.45%, 1.87 ± 0.33%, and 1.38 ± 0.40%, respectively (Fig. 3B). Further characterizing NK cells as a percentage of all CD45+/CD3−/CD19−/CD56+ cells (Fig. 3A, h) [36], we found that most of the cells were of the cytotoxic (CD56-positive, CD16high) phenotype (86.76 ± 0.90%) compared to the proliferative (CD56bright, CD16dim) phenotype (3.76 ± 0.36%) (Fig. 3C). The proportion of platelet adherence was also determined for each cell type (Fig. 3A, j–m). The percentage of platelet-positive individual cell type was quantified in Fig. 3D and shows that 2.10 ± 0.30% of T cells, 3.28 ± 0.40% of B cells, 2.40 ± 0.32% of NK cells, and 2.53 ± 0.39% of NKT cells had platelets adherent to the surface. Again, we used SEM to visualize immune cell-platelet aggregates and determined that platelets indeed adhere to the T-cells (Fig. 3E), NK cells (Fig. 3F) and NKT cells (Fig. 3G).

### Differences in blood cell composition between Caucasians and African Americans

Baseline characteristics of the 29 blood bank donors stratified by race/ethnicity are summarized in Additional file 1: Table S1. The data displayed in Figs. 1, 2 and 3 were further stratified by race/ethnicity, and some differences between the two groups were noted. African Americans (n = 14, median age 56.5  ±  17.5 years) were shown to have a lower proportion of total granulocytes (49.0% to 72.9%; p = 0.008) which most likely is the effect of having a lower proportion of neutrophils (48.61% to 63.95%; p = 0.023) and eosinophils (0.64% to 2.64%; p = 0.026) than Caucasians (n = 15, median age 60  ±  12.7 years), as shown in Table 2. No significant differences were observed in the basophil populations between these individuals. Table 2 also shows that African Americans had a greater proportion of total lymphocytes (33.75% to 16.7%; p = 0.008) than Caucasians; which can be attributed to greater proportions of T cells (25.15% to 12.25%; p = 0.009), B cells (2.08% to 1.02%; p = 0.0459), and NKT cells (0.87% to 0.34%; p = 0.046). No significant differences were seen in the NK cell (4.09% to 2.82%; p = 0.331) or total monocyte (7.59% to 7.96%; p = 0.644) populations between African American and Caucasian donors. Examining NK cell subset more closely (Table 2), African Americans were observed to have fewer cytotoxic NK cells (87.5% to 91.3%; p = 0.001; CD56dim/CD16high) than Caucasians, although no differences in proliferative NK cells (CD56high/CD16dim) were noted. While there were also no differences in the total monocyte population between the two groups, Table 2 shows that African Americans had fewer classical CD14^+^CD16^−^ monocytes (89.88% to 94.58%; p = 0.014) but higher proportions of intermediate CD14^+^CD16^+^ monocytes (5.74% to 2.61%; p = 0.01). No statistical difference was noted with respect to non-classical CD14^−^CD16^+^ monocytes. Table 2 shows that African Americans presented with a greater amount of platelet adhesion in non-classical monocytes (6.68% to 3.79%; p = 0.039) when compared to Caucasians, although no differences were observed in other monocyte, granulocyte, nor lymphocyte subsets. Stratification of the blood donor population by sex showed that basophil platelet aggregates were significantly higher in females (1.08% in males versus 3.85% in females, p = 0.03, Additional file 1: Table S2). There were no significant differences in cell or subset population distribution when the blood donor population was stratified by age (Additional file 1: Table S3).

**Table 2:** Percentage of all cell types in 500 µl of EDTA-heparinized whole blood stratified by ethnicity

	Caucasian (n = 15)	African-American (n = 14)	p-value
Median age	60 ± 12.7	56.5 ± 17.5	0.48
Granulocytes	72.9%	49.0%	0.008**
Neutrophils	63.95%	48.61%	0.02*
Eosinophils	2.64%	0.64%	0.03*
Basophils	0.07%	0.02%	0.06
Lymphocytes	16.7%	33.75%	0.008**
T cells	12.25%	25.15%	0.009**
B cells	1.02%	2.08%	0.05*
NKT cells	0.34%	0.87%	0.05*
NK cells	2.82%	4.09%	0.33
Proliferative NK cells (CD56^hi^CD16^dim^)	3.21%	3.50%	0.44
Cytotoxic NK cells (CD56^dim^/CD16^hi^)	91.3%	87.5%	0.001**
Monocytes	7.96%	7.59%	0.64
Classical monocytes (CD14^+^CD16^−^)	94.58%	89.88%	0.01*
Intermediate monocytes (CD14^+^CD16^+^)	2.61%	5.74%	0.01*
Non-classical monocytes (CD16^+^CD14^−^)	2.74%	5.04%	0.17
Platelet aggregates			
Neutrophils	2.54%	2.60%	0.50
Eosinophils	1.6%	0.96%	0.08
Basophils	3.25%	0.00%	0.10
T cells	1.97%	1.62%	0.29
B cells	2.96%	2.62%	0.45
NK cells	2.44%	1.67%	0.86
NKT cells	2.37%	1.2%	0.23
All monocytes	4.2%	2.39%	0.98
Classical monocytes (CD14^+^CD16^−^)	3.29%	2.01%	0.19
Intermediate monocytes (CD14^+^CD16^−^)	5.28%	3.20%	0.35
Non-classical monocytes (CD14^−^CD16^+^)	*3.79%*	*6.68%*	0.04*

Data are shown as median percentage of all single, live, CD45-positive cells. Results of subsets are shown as percentage of the parent gate (NK cells or monocytes). Statistical significance was determined after Mann-Whitney test. Significance is indicated by asterisk. Correspondent p-values are listed

Italic values indicate significant differences between the two groups

*p ≤ 0.05; **p < 0.01

### Association of flow cytometry-derived parameters with CVD risk markers

African American women with known CVD risk factors enrolled in a clinical protocol for cardiometabolic testing (N = 20) had their immune cell populations characterized using the described flow cytometry panel. The participants’ baseline characteristics are summarized in Additional file 1: Table S4, including Framingham Risk Score (FRS) as a measure of CVD risk. We examined the association between neutrophil counts based on the flow cytometry-derived measures and FRS in linear regression models adjusting for BMI. We compared these associations to the modeled associations between CBC-derived neutrophil counts and FRS. Both the CBC neutrophil count and the flow cytometry-derived neutrophil count were strongly associated with FRS in linear regression models adjusted for BMI (β = 0.49, p = 0.04 and 0.49, p = 0.04 respectively) (Table 3). Additionally, T cell proportions determined by flow cytometry associated with FRS in adjusted models (β = 0.47, p = 0.04). In models adjusting for BMI, T cell—(β = 0.59, p = 0.03), NK cell—(β = 0.55, p = 0.03), NKT cell—(β = 0.53, p = 0.03) and non-classical monocyte—(β = 0.54, p = 0.02) to-platelet aggregates were significantly associated with FRS. We also examined the association between hsCRP as a CVD risk marker [37, 38] and CBC or flow cytometry-derived cell counts in linear models adjusting for age and BMI. Within the patient population, immune cell-platelet aggregates correlated with hsCRP levels (Table 3). After adjustment for age and BMI, neutrophil- and eosinophil-platelet aggregates remained associated with hsCRP (β = 0.49, p = 0.04 and β = 0.49, p = 0.03, respectively). NK cell—platelet aggregates almost reached significance in adjusted models. No other significant associations with hsCRP were noted for flow cytometry-derived leukocyte populations.

**Table 3:** Linear regression models to demonstrate associations between clinical and flow cytometry derived cell populations and hsCRP as biomarker for CVD risk

	FRS β-coefficient (p-value)		hsCRP (mg/l) β-coefficient (p-value)	
	Unadjusted	Adj. for BMI	Unadjusted	Adj. for BMI/age
Clinical neutrophil population (%)	*0.49 (0.03)*	*0.49 (0.04)*	− 0.08 (0.73)	− 0.06 (0.79)
FCD neutrophil population (%)	*0.46 (0.04)*	*0.49 (0.04)*	0.08 (0.73)	− 0.04 (0.87)
Clinical eosinophil population (%)	− 0.05 (0.84)	− 0.05 (0.85)	− 0.04 (0.88)	0.16 (0.50)
FCD eosinophil population (%)	0.22 (0.36)	0.22 (0.36)	− 0.19 (0.43)	− 0.13 (0.58)
Clinical basophil population (%)	0.36 (0.12)	0.36 (0.14)	− 0.13 (0.59)	− 0.16 (0.49)
FCD basophil population (%)	0.20 (0.39)	0.21 (0.39)	− 0.24 (0.31)	− 0.18 (0.45)
Clinical monocyte population (%)	0.38 (0.097)	0.38 (0.11)	− 0.35 (0.13)	− 0.37 (0.10)
FCD monocyte population (%)	− 0.20 (0.40)	− 0.20 (0.42)	− 0.36 (0.12)	− 0.32 (0.15)
Clinical lymphocyte population (%)	− *0.53 (0.02)*	− *0.55 (0.02)*	0.18 (0.45)	0.10 (0.66)
FCD T cell population (%)	− *0.47 (0.04)*	− *0.47 (0.04)*	0.10 (0.68)	0.14 (0.57)
FCD B cell population (%)	− 0.196 (0.41)	− 0.20 (0.42)	0.18 (0.44)	0.35 (0.18)
FCD NK cell population (%)	− 0.085 (0.72)	− 0.09 (0.72)	− 0.07 (0.77)	− 0.15 (0.52)
FCD NKT cell population (%)	− 0.020 (0.93)	− 0.02 (0.94)	0.22 (0.27)	0.28 (0.20)
FCD cell-platelet aggregate (PA)				
Neutrophils-PA	0.18 (0.45)	0.21 (0.44)	*0.58 (0.01)*	*0.49 (0.04)*
Eosinophils-PA	0.20 (0.39)	0.22 (0.39)	*0.58 (0.01)*	*0.49 (0.03)*
Basophils-PA	0.13 (0.58)	0.13 (0.60)	0.22 (0.36)	0.17 (0.46)
T cells-PA	*0.45 (0.05)*	*0.59 (0.03)*	*0.52 (0.02)*	0.41 (0.11)
B cells-PA	0.27 (0.24)	0.35 (0.20)	*0.46 (0.04)*	0.31 (0.21)
NK cells-PA	0.44 (0.05)	*0.55 (0.03)*	*0.55 (0.01)*	0.45 (0.07)
NKT cells-PA	0.43 (0.06)	*0.53 (0.04)*	*0.45 (0.04)*	0.32 (0.20)
All monocyte-PA	0.24 (0.32)	0.24 (0.33)	0.23 (0.33)	0.19 (0.39)
Classical monocyte-PA	0.19 (0.42)	0.19 (0.43)	0.22 (0.34)	0.19 (0.39)
Intermediate monocyte-PA	0.18 (0.44)	0.18 (0.46)	0.23 (0.34)	0.23 (0.32)
Nonclassical monocyte-PA	*0.52 (0.02)*	*0.54 (0.02)*	0.28 (0.23)	0.20 (0.40)

Unadjusted and adjusted regressions (for age and BMI) were performed between the indicated parameters for African American women (N = 20) at increased risk for CVD. Each regression is given as β-coefficient with the p-value in parenthesis

Significant associations are italicized

*FCD* flow cytometry derived

## Discussion

We have developed a flow cytometry panel that allows for characterization of granulocyte, monocyte, and lymphocyte populations implicated in CVD using 500 µl of blood. Data obtained using this flow cytometry panel, in conjunction with microscopy data, demonstrate the feasibility of the method. Our initial data also highlight racial/ethnic differences in immune cell populations between African Americans and Caucasians which we hypothesize may contribute to increased CVD risk in African American populations; however, these findings must be replicated in larger cohorts. With this technique, scientists from a broad range of disciplines who may normally rely on the CBC from clinical specimens to characterize immune and hematopoietic cell populations can quantify a large number of CVD-implicated immune cell types using 500 µl of blood. This low-blood volume flow cytometry panel provides several advantages over the CBC. The CBC provides information on overall red and white blood cell count as well as a general platelet count, but requires staining techniques and microscopy analysis for determining cellular subpopulations in human blood [39]. Moreover, the CBC cannot account for specific markers and their expression levels on subpopulations of white blood cells, nor will it allow the identification and quantification of different monocyte subpopulations or NK cells. Also, the CBC cannot account for ‘hitchhiking’ platelets, which are associated with CVD risk [31, 40–42], especially highlighted in a recent study focusing on women [43].

Many of the leukocytic differences that we have presented have been reaffirmed in the literature, supporting the validity of our flow cytometry panel. African American blood donors have been shown to have decreased levels of circulating neutrophils, and this phenomenon has been described as benign ethnic neutropenia [44]. Circulating amounts of lymphocytes have also been shown to be increased in African Americans [45]. In a vaccination study, African Americans were shown to have a greater proportion of B cells than Caucasians at baseline [46]. Moreover, it has been demonstrated that African Americans have fewer classical, more intermediate, and more non-classical monocytes than Caucasians [47]. To our knowledge, increased levels of NKT cells, decreased levels of cytotoxic NK cells, and the observed increases in non-classical monocyte platelet adhesion in African Americans have not been previously demonstrated. An increased presence of intermediate and non-classical monocytes are known to accelerate heart disease [48, 49] while increased monocyte-platelet adhesion has been described as an early marker for acute myocardial infarction [50]. Moreover, NKT cells have been shown to have atherogenic properties [51], specifically through the activation of the CD1d receptor by hyperlipidemic conditions [52]. Cytotoxic NK cells and NKT cells have also been observed to be decreased in coronary artery disease patients when compared to healthy controls [53]. These leukocytic shifts may relate to greater CVD risk among African Americans as compared to Caucasians. But the findings related to NKT cells, NK cells and monocytes must be further explored in larger cohorts and assessed in relation to specific CVD risk factors like hypertension, obesity, and diabetes, which are more prevalent among African Americans [2]. Our findings are supported by prior work demonstrating an association between leukocyte counts, particularly neutrophil counts, and CVD risk factors in a large population-based cohort [54]. These findings suggest that the flow-derived measures may also serve as potential markers in CV risk prediction; however, this flow panel must be studied in larger, diverse populations with well-characterized CVD outcomes. We do acknowledge that the healthy blood bank donors included in our study could not be characterized further than race/ethnicity, sex, and age, which does limit our results because we are unable to account for the impact of health factors, including BMI, comorbidities, health behaviors (i.e. dietary intake, physical activity, smoking status), and/or socio-demographic factors that may contribute to CVD risk, in our results.

Importantly, we aimed to validate our findings by demonstrating correlation of flow-derived findings with FRS and hsCRP. Recent literature supports the existence and importance of immune cell-to-platelet aggregates in CVD [40–42, 55, 56]. Interestingly, the quality and quantity of platelet adhesion differs between the various immune cells [57, 58]. Very recently, Barrett et al demonstrated that platelet adhesion on monocytes in mice promoted an inflammatory and pro-atherogenic phenotype accompanied by increased leukocyte trafficking and macrophage accumulation within the atherosclerotic plaque [43]. Utilizing human samples, Barrett et al. also showed a positive correlation of monocyte-platelet aggregates with atherosclerosis severity in two cohorts, including a cohort of women with or without myocardial infarction. This recently published manuscript further strengthens the importance of monocyte-to-platelet adhesion in myocardial infarction and provides support for immune cell characterization beyond CBC, especially immune cell-platelet aggregates. Our data show that after adjustment, immune cell–platelet aggregates associates with increasing FRS, while the CBC platelet count did not show a significant association. Certainly, larger studies are needed; however, our data suggest, that detection of platelet-aggregates in the clinical setting might also be useful in determining CVD risk.

By developing a flow cytometry technique that allows immune cell phenotyping with 500 µl of blood, we are able to minimize the blood sample required and gather a large amount of phenotypic data. There is potential for broad applicability of this flow cytometry panel in cardiovascular research. For instance, as more CVD therapies target immune cell function [8], assays like this flow cytometry panel may be useful to assess immune response to drug therapy with limited blood volume; this will be especially important in the era of precision medicine where blood samples are already needed for genotyping and deep phenotyping of clinical biomarkers [59]. This panel is minimally invasive and may also be especially applicable in health disparities research focused on CVD. Populations recruited to studies addressing health disparities are typically underrepresented in clinical research and may be wary of providing larger volumes of blood due to limited trust of the scientific community with its history of inequitable treatment and unethical experimentation [60]. Future studies to address health disparities could employ these assays in diverse populations to assess potential mechanisms by which differential exposure to adverse psychosocial or environmental conditions and racial/ethnic differences in the inflammatory response and hematopoiesis combine to contribute to poor CVD outcomes for those living with heightened CVD risk [6]. It has to be emphasized that depending on the available flow cytometer and user-dependent familiarity with flow cytometry many more markers can be added to this panel to further characterize immune cells. For example, by adding various T cell markers, T cell subsets like CD4 or CD8 or more specific subsets like Th1 or Th17 cells could be further characterized. Also, the identification of neutrophil subsets [31] or stem and progenitor cells could be achieved by adding additional markers.

Prior efforts have been made to standardize and possibly even automate flow cytometry analysis of blood cells, especially when large cohorts are involved, and these methods come with their own advantages and disadvantages, as summarized previously [61, 62]. For example, the use of Lyoplates (BD Biosciences, USA) had been suggested for use in standardized identification of cell surface markers on PBMCs [63]. However, larger screens using the full panel of Lyoplates generally require a larger volume of donor blood than 500 µl. Additionally, the use of Lyoplates is usually associated with high cost. Therefore, researchers should identify the goals of their study and weigh the amount of study participant blood requested, cost per study participant, and amount of time needed to analyze large datasets.

While an advantage of flow cytometry is the variety of available antibodies and fluorochrome combinations, there are some potential limitations of flow cytometry to consider. Researchers often rely on the information published in the literature when choosing the appropriate clone and antibody. Ideally, a variety of clones of one antigen should be tested beforehand and the literature searched in order to find the suitable clone for the individual study design. Additionally, years of antibody production could cause clones to lose antigen recognition or present difficulties in data reproducibility [62, 64]. The computational data analysis of each sample using flow cytometry will take time and should also be carried out cautiously. Additionally, compensation and titration runs should be repeated whenever new batches of antibodies are obtained or the flow cytometer utilized undergoes repair and maintenance. Another important limitation of the presented flow cytometry protocol is the inability to provide a specific cell count per microliter of blood. As such, results from this technique must be shown as ratios from all non-debris, single, live, and CD45-positive cells. A benefit of using the differential blood count is that it provides a specific cell count per microliter of blood per cell type and is used commonly to identify leukocytosis, which may be indicative of infection [65].

## Conclusion

In summary, the low-blood volume flow cytometry panel can be an efficient method to phenotype immune cell populations associated with CVD using only 500 µl of blood. Because this panel only requires a small volume of blood, it may have wide-ranging applicability in cardiovascular research as future studies require more detailed characterization of immune cells.

## Supplementary information

**Additional file 1.** Additional figures and tables.

## Data Availability

Data can be requested from the corresponding author upon reasonable request.

## Abbreviations

AA: African American
CD: cluster of differentiation
CVD: cardiovascular disease
SEM: scanning electron microscopy
SES: socioeconomic status
NK cells: Natural killer cells
NKT cells: Natural killer T cells
PBMC: peripheral blood mononuclear cells
FRS: Framingham risk score
hsCRP: high sensitivity c-reactive protein

## References

[1] Benjamin EJ, Blaha MJ, Chiuve SE, Cushman M, Das SR, Deo R, de Ferranti SD, Floyd J, Fornage M, Gillespie C (2017). Heart disease and stroke statistics-2017 update: a report from the American Heart Association. Circulation.

[2] Carnethon MR, Pu J, Howard G, Albert MA, Anderson CAM, Bertoni AG, Mujahid MS, Palaniappan L, Taylor HA, Willis M (2017). Cardiovascular Health in African Americans: a Scientific Statement From the American Heart Association. Circulation.

[3] Saab KR, Kendrick J, Yracheta JM, Lanaspa MA, Pollard M, Johnson RJ (2015). New insights on the risk for cardiovascular disease in African Americans: the role of added sugars. J Am Soc Nephrol.

[4] Thomas AJ, Eberly LE, Davey Smith G, Neaton JD, Stamler J (2005). Race/ethnicity, income, major risk factors, and cardiovascular disease mortality. Am J Public Health.

[5] Lei MK, Beach SRH, Simons RL (2018). Biological embedding of neighborhood disadvantage and collective efficacy: influences on chronic illness via accelerated cardiometabolic age. Dev Psychopathol.

[6] Bagby SP, Martin D, Chung ST, Rajapakse N (2019). From the outside in: biological mechanisms linking social and environmental exposures to chronic disease and to health disparities. Am J Public Health.

[7] Gistera A, Hansson GK (2017). The immunology of atherosclerosis. Nat Rev Nephrol.

[8] Ridker PM, Everett BM, Thuren T, MacFadyen JG, Chang WH, Ballantyne C, Fonseca F, Nicolau J, Koenig W, Anker SD (2017). Antiinflammatory therapy with canakinumab for atherosclerotic disease. N Engl J Med.

[9] Chavez-Sanchez L, Espinosa-Luna JE, Chavez-Rueda K, Legorreta-Haquet MV, Montoya-Diaz E, Blanco-Favela F (2014). Innate immune system cells in atherosclerosis. Arch Med Res.

[10] Boisvert WA (2001). The participation of inflammatory cells in atherosclerosis. Drugs Today (Barc).

[11] Woollard KJ, Geissmann F (2010). Monocytes in atherosclerosis: subsets and functions. Nat Rev Cardiol.

[12] Schaftenaar F, Frodermann V, Kuiper J, Lutgens E (2016). Atherosclerosis: the interplay between lipids and immune cells. Curr Opin Lipidol.

[13] Ilhan F, Kalkanli ST (2015). Atherosclerosis and the role of immune cells. World J Clin Cases.

[14] Getz GS, Reardon CA (2017). Natural killer T cells in atherosclerosis. Nat Rev Cardiol.

[15] Doring Y, Soehnlein O, Weber C (2017). Neutrophil extracellular traps in atherosclerosis and atherothrombosis. Circ Res.

[16] Doring Y, Drechsler M, Soehnlein O, Weber C (2015). Neutrophils in atherosclerosis: from mice to man. Arterioscler Thromb Vasc Biol.

[17] Cochain C, Zernecke A (2017). Macrophages in vascular inflammation and atherosclerosis. Pflugers Arch.

[18] Cochain C, Zernecke A (2015). Macrophages and immune cells in atherosclerosis: recent advances and novel concepts. Basic Res Cardiol.

[19] Jaiswal S, Natarajan P, Silver AJ, Gibson CJ, Bick AG, Shvartz E, McConkey M, Gupta N, Gabriel S, Ardissino D (2017). Clonal hematopoiesis and risk of atherosclerotic cardiovascular disease. N Engl J Med.

[20] Natarajan P, Jaiswal S, Kathiresan S (2018). Clonal hematopoiesis: somatic mutations in blood cells and atherosclerosis. Circ Genom Precis Med.

[21] Fuster JJ, Walsh K (2018). Somatic mutations and clonal hematopoiesis: unexpected potential new drivers of age-related cardiovascular disease. Circ Res.

[22] Sano S, Oshima K, Wang Y, MacLauchlan S, Katanasaka Y, Sano M, Zuriaga MA, Yoshiyama M, Goukassian D, Cooper MA (2018). Tet2-mediated clonal hematopoiesis accelerates heart failure through a mechanism involving the IL-1beta/NLRP3 inflammasome. J Am Coll Cardiol.

[23] Libby P (2017). Interleukin-1 beta as a target for atherosclerosis therapy: biological basis of CANTOS and beyond. J Am Coll Cardiol.

[24] Hilgendorf I, Swirski FK, Robbins CS (2015). Monocyte fate in atherosclerosis. Arterioscler Thromb Vasc Biol.

[25] Bonaccorsi I, De Pasquale C, Campana S, Barberi C, Cavaliere R, Benedetto F, Ferlazzo G (2015). Natural killer cells in the innate immunity network of atherosclerosis. Immunol Lett.

[26] Libby P, Hansson GK (2015). Inflammation and immunity in diseases of the arterial tree: players and layers. Circ Res.

[27] Soehnlein O (2012). Multiple roles for neutrophils in atherosclerosis. Circ Res.

[28] Libby P (2012). Inflammation in atherosclerosis. Arterioscler Thromb Vasc Biol.

[29] Braun NA, Covarrubias R, Major AS (2010). Natural killer T cells and atherosclerosis: form and function meet pathogenesis. J Innate Immun.

[30] Baumer Y, Ng Q, Sanda GE, Dey AK, Teague HL, Sorokin AV, Dagur PK, Silverman JI, Harrington CL, Rodante JA (2018). Chronic skin inflammation accelerates macrophage cholesterol crystal formation and atherosclerosis. JCI Insight.

[31] Teague HL, Varghese NJ, Tsoi LC, Dey AK, Garshick MS, Silverman JI, Baumer Y, Harrington CL, Stempinski E, Elnabawi YA (2019). Neutrophil subsets, platelets, and vascular disease in psoriasis. JACC Basic Transl Sci.

[32] Baumer Y, McCurdy S, Weatherby TM, Mehta NN, Halbherr S, Halbherr P, Yamazaki N, Boisvert WA (2017). Hyperlipidemia-induced cholesterol crystal production by endothelial cells promotes atherogenesis. Nat Commun.

[33] Baumer Y, McCurdy S, Alcala M, Mehta N, Lee BH, Ginsberg MH, Boisvert WA (2017). CD98 regulates vascular smooth muscle cell proliferation in atherosclerosis. Atherosclerosis.

[34] Jahangiry L, Farhangi MA, Rezaei F (2017). Framingham risk score for estimation of 10-years of cardiovascular diseases risk in patients with metabolic syndrome. J Health Popul Nutr.

[35] Dagur PK, McCoy JP (2015). Collection, storage, and preparation of human blood cells. Curr Protoc Cytom.

[36] Poli A, Michel T, Theresine M, Andres E, Hentges F, Zimmer J (2009). CD56bright natural killer (NK) cells: an important NK cell subset. Immunology.

[37] Musunuru K, Kral BG, Blumenthal RS, Fuster V, Campbell CY, Gluckman TJ, Lange RA, Topol EJ, Willerson JT, Desai MY (2008). The use of high-sensitivity assays for C-reactive protein in clinical practice. Nat Clin Pract Cardiovasc Med.

[38] Mora S, Musunuru K, Blumenthal RS (2009). The clinical utility of high-sensitivity C-reactive protein in cardiovascular disease and the potential implication of JUPITER on current practice guidelines. Clin Chem.

[39] Blumenreich MS, Walker HK, Hall WD, Hurst JW (1990). The white blood cell and differential count. Clinical methods: the history, physical, and laboratory examinations.

[40] Wrigley BJ, Shantsila E, Tapp LD, Lip GY (2013). Increased formation of monocyte-platelet aggregates in ischemic heart failure. Circ Heart Fail.

[41] Gregg D, Goldschmidt-Clermont PJ (2003). Cardiology patient page. Platelets and cardiovascular disease. Circulation.

[42] Ashman N, Macey MG, Fan SL, Azam U, Yaqoob MM (2003). Increased platelet-monocyte aggregates and cardiovascular disease in end-stage renal failure patients. Nephrol Dial Transplant.

[43] Barrett TJ, Schlegel M, Zhou F, Gorenchtein M, Bolstorff J, Moore KJ, Fisher EA, Berger JS (2019). Platelet regulation of myeloid suppressor of cytokine signaling 3 accelerates atherosclerosis. Sci Transl Med.

[44] Hsieh MM, Everhart JE, Byrd-Holt DD, Tisdale JF, Rodgers GP (2007). Prevalence of neutropenia in the U.S. population: age, sex, smoking status, and ethnic differences. Ann Intern Med.

[45] Bain BJ (1996). Ethnic and sex differences in the total and differential white cell count and platelet count. J Clin Pathol.

[46] Kurupati R, Kossenkov A, Haut L, Kannan S, Xiang Z, Li Y, Doyle S, Liu Q, Schmader K, Showe L, Ertl H (2016). Race-related differences in antibody responses to the inactivated influenza vaccine are linked to distinct pre-vaccination gene expression profiles in blood. Oncotarget.

[47] Appleby LJ, Nausch N, Midzi N, Mduluza T, Allen JE, Mutapi F (2013). Sources of heterogeneity in human monocyte subsets. Immunol Lett.

[48] Wildgruber M, Aschenbrenner T, Wendorff H, Czubba M, Glinzer A, Haller B, Schiemann M, Zimmermann A, Berger H, Eckstein HH (2016). The “Intermediate” CD14(++)CD16(+) monocyte subset increases in severe peripheral artery disease in humans. Sci Rep.

[49] Zungsontiporn N, Tello RR, Zhang G, Mitchell BI, Budoff M, Kallianpur KJ, Nakamoto BK, Keating SM, Norris PJ, Ndhlovu LC (2016). Non-classical monocytes and monocyte chemoattractant protein-1 (MCP-1) correlate with coronary artery calcium progression in chronically HIV-1 infected adults on stable antiretroviral therapy. PLoS ONE.

[50] Furman MI, Barnard MR, Krueger LA, Fox ML, Shilale EA, Lessard DM, Marchese P, Frelinger AL, Goldberg RJ, Michelson AD (2001). Circulating monocyte-platelet aggregates are an early marker of acute myocardial infarction. J Am Coll Cardiol.

[51] van Puijvelde GHM, Kuiper J (2017). NKT cells in cardiovascular diseases. Eur J Pharmacol.

[52] Aslanian AM, Chapman HA, Charo IF (2005). Transient role for CD1d-restricted natural killer T cells in the formation of atherosclerotic lesions. Arterioscler Thromb Vasc Biol.

[53] Jabir NR, Firoz CK, Ahmed F, Kamal MA, Hindawi S, Damanhouri GA, Almehdar HA, Tabrez S (2017). Reduction in CD16/CD56 and CD16/CD3/CD56 natural killer cells in coronary artery disease. Immunol Invest.

[54] Yen ML, Yang CY, Yen BL, Ho YL, Cheng WC, Bai CH (2006). Increased high sensitivity C-reactive protein and neutrophil count are related to increased standard cardiovascular risk factors in healthy Chinese men. Int J Cardiol.

[55] Lam FW, Vijayan KV, Rumbaut RE (2015). Platelets and their interactions with other immune cells. Compr Physiol.

[56] da Costa Martins P, van den Berk N, Ulfman LH, Koenderman L, Hordijk PL, Zwaginga JJ (2004). Platelet-monocyte complexes support monocyte adhesion to endothelium by enhancing secondary tethering and cluster formation. Arterioscler Thromb Vasc Biol.

[57] Li N, Ji Q, Hjemdahl P (2006). Platelet-lymphocyte conjugation differs between lymphocyte subpopulations. J Thromb Haemost.

[58] Ahn KC, Jun AJ, Pawar P, Jadhav S, Napier S, McCarty OJ, Konstantopoulos K (2005). Preferential binding of platelets to monocytes over neutrophils under flow. Biochem Biophys Res Commun.

[59] Shah SH, Arnett D, Houser SR, Ginsburg GS, MacRae C, Mital S, Loscalzo J, Hall JL (2016). Opportunities for the cardiovascular community in the precision medicine initiative. Circulation.

[60] Byrd WM, Clayton LA (2001). Race, medicine, and health care in the United States: a historical survey. J Natl Med Assoc.

[61] Maecker HT, McCoy JP, Nussenblatt R (2012). Standardizing immunophenotyping for the human immunology project. Nat Rev Immunol.

[62] Bradbury A, Pluckthun A (2015). Reproducibility: standardize antibodies used in research. Nature.

[63] Villanova F, Di Meglio P, Inokuma M, Aghaeepour N, Perucha E, Mollon J, Nomura L, Hernandez-Fuentes M, Cope A, Prevost AT (2013). Integration of lyoplate based flow cytometry and computational analysis for standardized immunological biomarker discovery. PLoS ONE.

[64] Baker M (2015). Reproducibility crisis: blame it on the antibodies. Nature.

[65] Abramson N, Melton B (2000). Leukocytosis: basics of clinical assessment. Am Fam Physician.

