# Supplementary material for: Immune cell phenotyping in low blood volumes for assessment of cardiovascular disease risk, development, and progression: a pilot study
Source: J Transl Med. 2020 Jan 17;18:29. doi: 10.1186/s12967-020-02207-0 (PMC6966880; doi:10.1186/s12967-020-02207-0)
Supplement: Supplementary file 1 — Additional file 1. Additional figures and tables. [file 12967_2020_2207_MOESM1_ESM.docx]

**Additional Figures:**


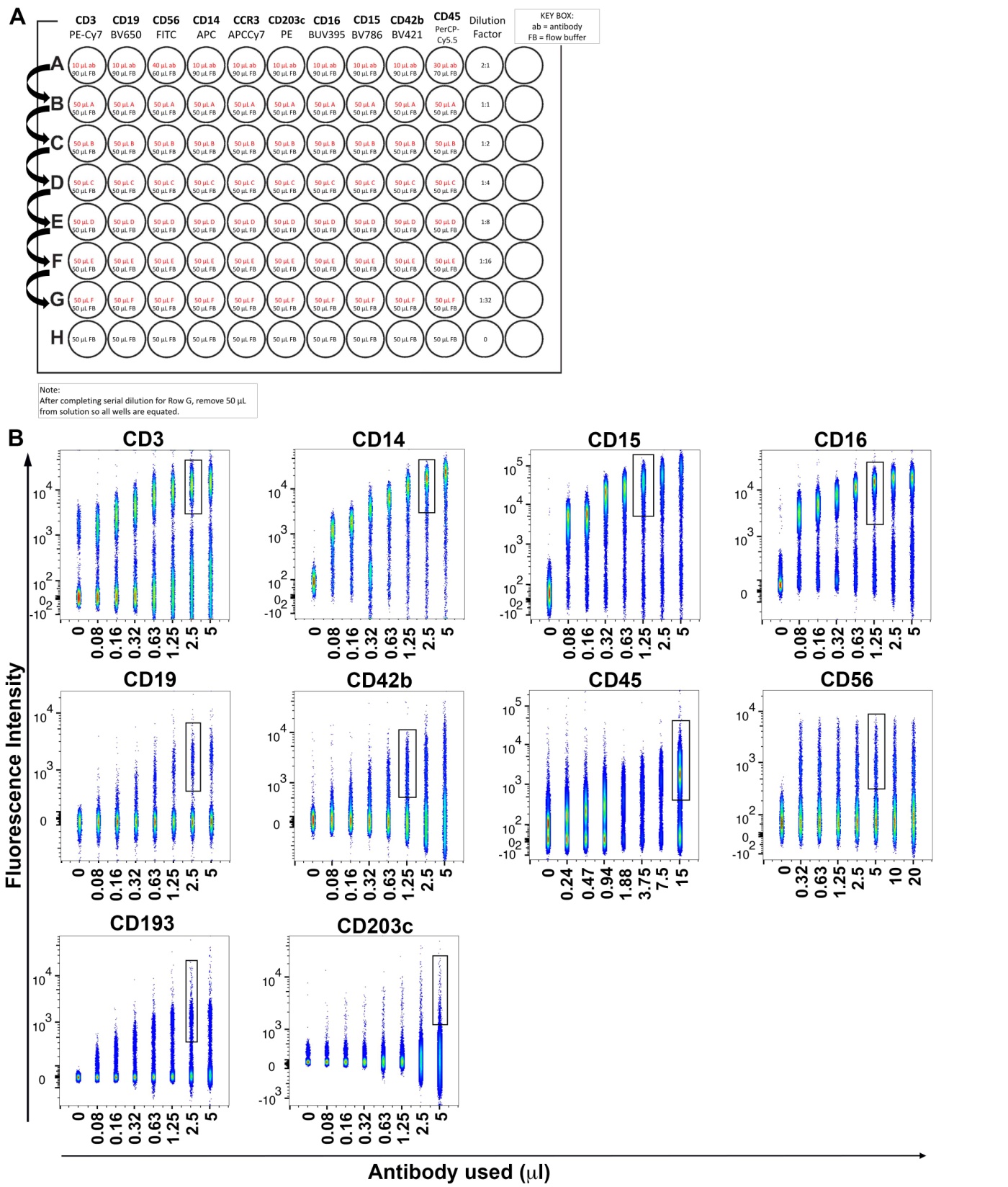


**Figure S1: Determination of ideal antibody amount needed to obtain sufficient labeling of target cells.** (**A**) Whole blood cells were prepared and added to a 96-well round bottom plate with serial antibody dilutions present as shown in (**A**). (**B**) Representative blots are shown for each antibody displaying the dilution on the x-axis (given in μl per antibody) and the detected fluorescence in each individual laser on the y-axis. Ideal dilution is indicated for each individual antibody. (ab-antibody, FB-flow buffer)


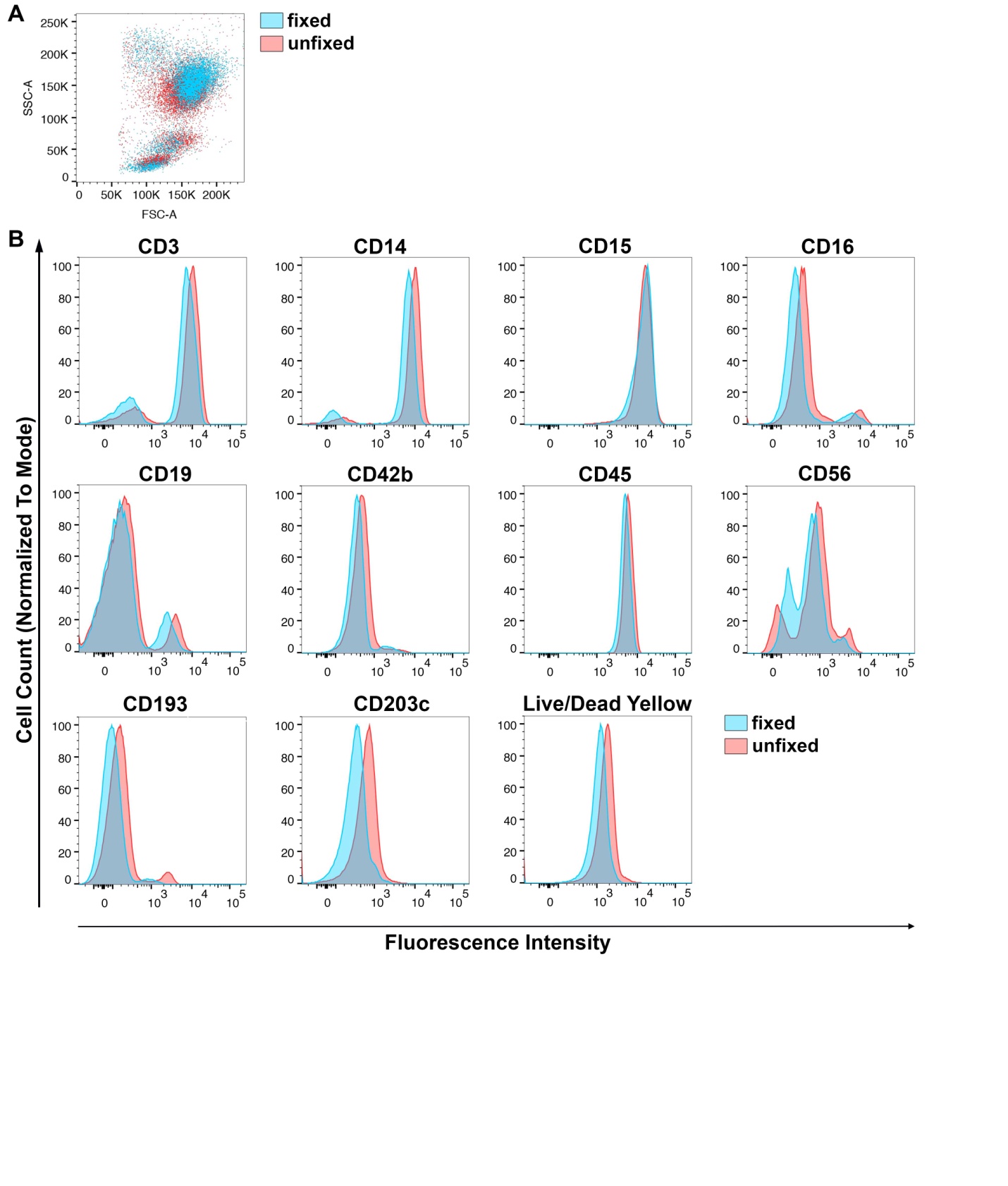


**Figure S2: Determining the impact of fixation on whole blood staining with subsequent flow cytometry analysis.** (**A**) Displayed is the overlay scatter blot of whole blood cells after staining with and without fixation. (**B**) Representative histograms for each antibody are shown overlaying fixed (blue) and unfixed (red) samples.


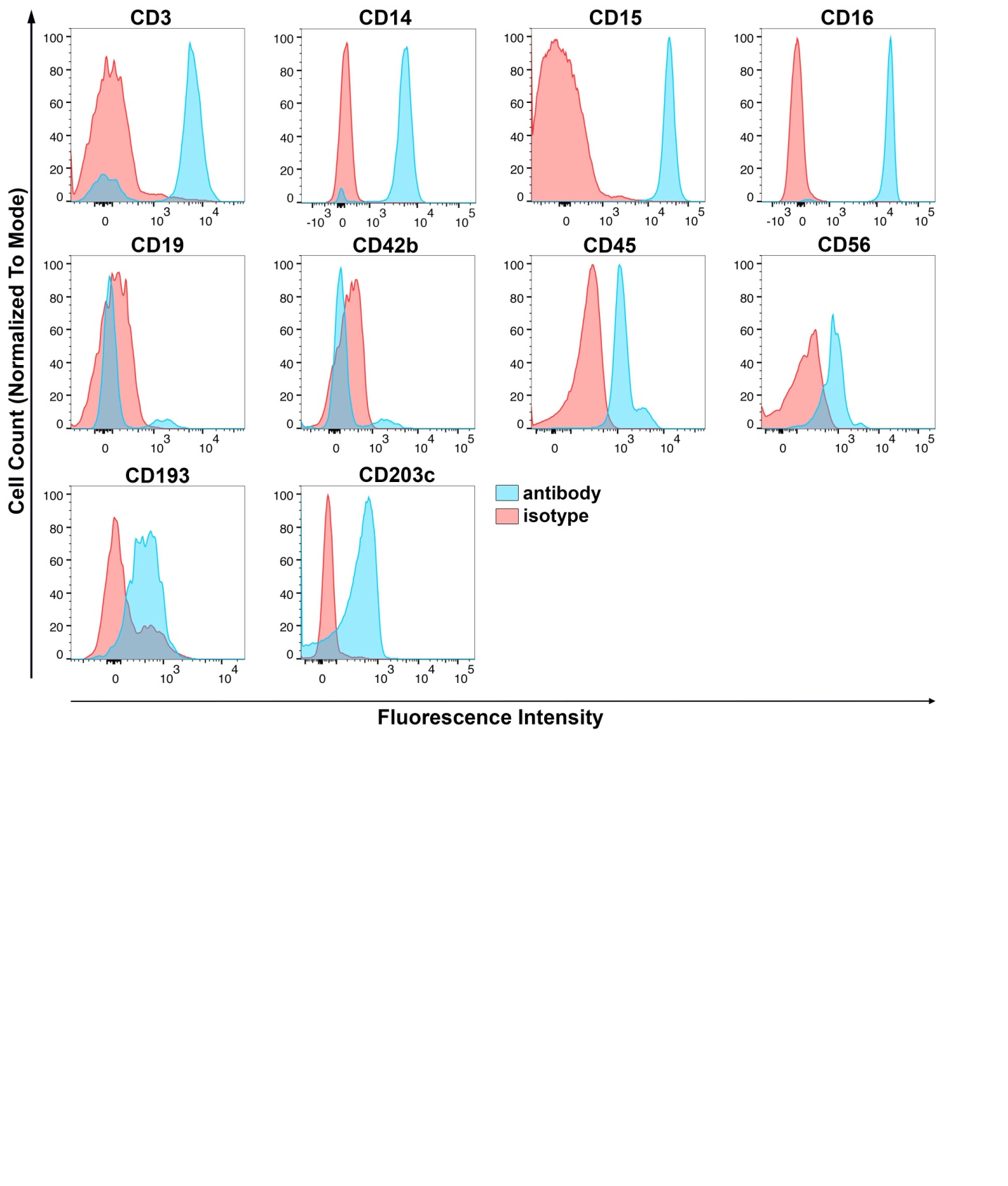


**Figure S3: Detection of nonspecific antibody binding.** Cells from whole blood were prepared as described and stained with the antibodies and their respective fluorescence-tagged isotype controls to display a distinct separation of isotype control and actual antibody staining in the histogram blots. Histograms of each antibody (blue) and isotype control (red) are shown and derived from the specific parent gate.


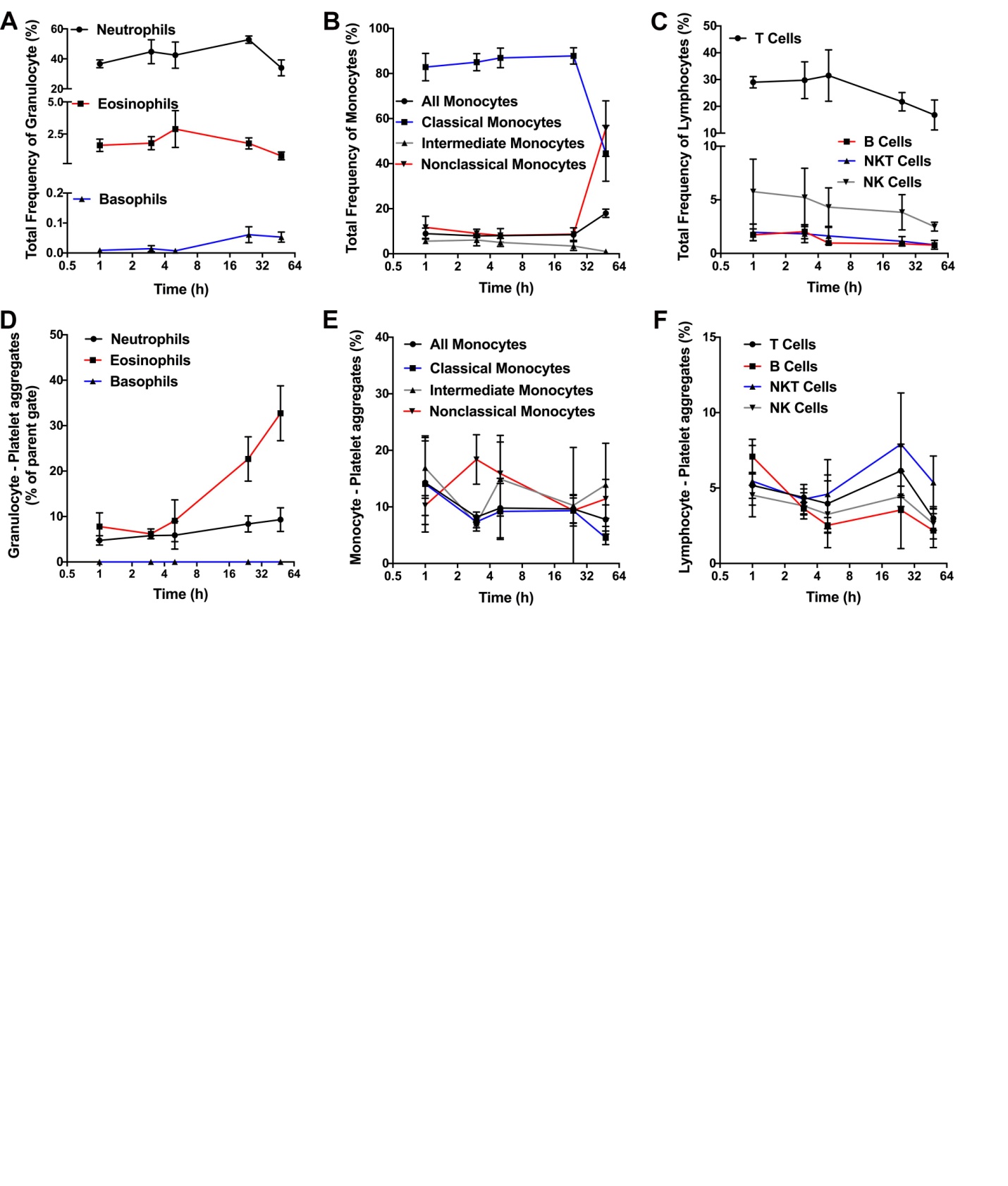


**Figure S4: Time-laps experiment to assess stability of obtained data during blood storage.** Heparinized blood was drawn from 3 blood bank donors and subjected to immunophenotyping at various time points (1h, 3h, 5h, 24h and 48h after drawing). Timepoints were taken from a green top tube under sterile conditions. Optimal results are obtained within 5h of the blood being drawn.

**Additional Tables:**

**Table S1: Summary of blood bank donor participant demographics**. 29 health blood bank donors were used in the first part of this manuscript. The basic available demographics of these donors are summarized in this table.

|  | **Caucasians (N=15)** | **African Americans (N=14)** |
| --- | --- | --- |
| **Female Sex, N (%)** | 7 (46.7%) | 10 (71.4%) |
| **Age (years)** | 60.0 ± 12.7 | 56.5 ± 17.5 |

**Table S2: Stratification of the results from 29 healthy blood bank donors by sex.** Results for sex are shown male vs female.

|  |  | Male  (N=12) | Female  (N=17) | p-value |
| --- | --- | --- | --- | --- |
| Median age |  | 57 ± 16.9 | 60 ± 13.4 | 0.93 |
| Granulocytes |  | 69.04% | 54.51% | 0.42 |
| Neutrophils |  | 63.59% | 50.07% | 0.51 |
| Eosinophils |  | 5.39% | 4.29% | 0.51 |
| Basophils |  | 0.06% | 0.15% | 0.33 |
| Lymphocytes |  | 17.34% | 25.90% | 0.54 |
| T cells |  | 12.59% | 20.21% | 0.93 |
| B cells |  | 1.09% | 1.51% | 0.40 |
| NKT cells |  | 0.56% | 0.99% | 0.51 |
| NK cells |  | 3.10% | 3.19% | 0.96 |
| Proliferative NK cells (CD56^hi^/CD16dim) | | 3.43% | 3.47% | 0.93 |
| Cytotoxic NK cells (CD56^dim^/CD16^hi^) | | 90.9% | 87.8% | 0.20 |
| Monocytes |  | 8.10% | 9.76% | 0.27 |
| Classical monocytes (CD14^+^CD16^-^) | | 88.44% | 90.10% | 0.72 |
| Intermediate monocytes (CD14^+^CD16^+^) | | 4.19% | 4.89% | 0.60 |
| Non-classical monocytes (CD16^+^CD14^-^) | | 6.25% | 5.07% | 0.38 |
| Platelet aggregates | | | | |
| Neutrophils | | 2.42% | 2.07% | 0.66 |
| Eosinophils | | 1.31% | 1.56% | 0.35 |
| Basophils | | **1.08%** | **3.85%** | **0.03*** |
| T cells | | 1.62% | 1.38% | 0.93 |
| B cells | | 2.81% | 2.76% | 0.76 |
| NK cells | | 2.91% | 1.26% | 0.25 |
| NKT cells | | 1.28% | 2.07% | 0.54 |
| All monocytes | | 2.405% | 1.98% | 0.45 |
| Classical monocytes (CD14^+^CD16^-^) | | 2.33% | 1.99% | 0.40 |
| Intermediate monocytes (CD14^+^CD16^-^) | | 2.90% | 2.75% | 0.67 |
| Non-classical monocytes (CD14^-^CD16^+^) | | 3.96% | 3.17% | 0.82 |

**Table S3: Stratification of the results from healthy blood bank donors by age.** For 23 of the 29 healthy blood bank donors the age was available, and the data stratified by age (below and above 60 years).

|  |  | Age < 60 years (N=12) | Age ≥ 60 years (N=11) | p-value |
| --- | --- | --- | --- | --- |
| Median Age |  | 51.5 ± 11.4 | 66 ± 8.2 | **0.00001** |
| Granulocytes |  | 59.78% | 69.33% | 0.59 |
| Neutrophils |  | 55.17% | 65.55% | 0.36 |
| Eosinophils |  | 4.56% | 3.72% | 0.90 |
| Basophils |  | 0.05% | 0.06% | 0.50 |
| Lymphocytes |  | 26.47% | 16.07% | 0.48 |
| T cells |  | 20.91% | 11.95% | 0.20 |
| B cells |  | 1.90% | 0.89% | 0.24 |
| NKT cells |  | 0.68% | 0.39% | 0.81 |
| NK cells |  | 2.98% | 2.85% | 0.67 |
| Proliferative NK cells (CD56^hi^CD16^dim^) | | 3.55% | 3.63% | 1.00 |
| Cytotoxic NK cells (CD56^dim^/CD16^hi^) | | 87.25% | 88.4% | 0.37 |
| Monocytes |  | 8.51% | 7.37% | 0.46 |
| Classical monocytes (CD14^+^CD16^-^) | | 88.13% | 90.10% | 0.72 |
| Intermediate monocytes (CD14^+^CD16^+^) | | 4.87% | 3.95% | 0.95 |
| Non-classical monocytes (CD16^+^CD14^-^) | | 5.95% | 5.07% | 0.71 |
| Platelet aggregates | | | | |
| Neutrophils | | 1.81% | 2.07% | 0.62 |
| Eosinophils | | 1.27% | 1.46% | 0.73 |
| Basophils | | 1.08% | 2.67% | 0.35 |
| T cells | | 1.16% | 1.45% | 0.93 |
| B cells | | 2.47% | 2.03% | 0.62 |
| NK cells | | 2.22% | 1.75% | 0.58 |
| NKT cells | | 1.55% | 1.33% | 0.67 |
| All monocytes | | 2.27% | 2.17% | 0.95 |
| Classical monocytes (CD14^+^CD16^-^) | | 2.26% | 2.08% | 0.67 |
| Intermediate monocytes (CD14^+^CD16^-^) | | 2.17% | 2.55% | 0.67 |
| Non-classical monocytes (CD14^-^CD16^+^) | | 3.55% | 3.07% | 0.58 |

**Table S4:** Summary of study participants. Values reported in the table as Mean ± SD or Median (IQR) for continuous data and N (%) for categorical data.

| **Participants’ Demographics and clinical parameters** | **Study participants (n=20)** |
| --- | --- |
| Gender, Female | 20 (100) |
| Ethnicity, African-American | 20 (100) |
| Age, years | 59.6 ± 12.4 |
| BMI, kg/m^2^ | 33.9 ± 7.6 |
| Systolic Blood Pressure (SBP), mmHg | 133.1 ± 14.1 |
| Diastolic Blood Pressure (DBP), mmHg | 74.9 ± 10.2 |
| Cholesterol, mg/dl | 200.2 ± 36.2 |
| HDL, mg/dl | 69.5 ± 27.6 |
| LDL, mg/dl | 111.7 ± 37.4 |
| Triglycerides, mg/dl | 95.2 ± 27.3 |
| hsCRP, mg/l | 6.2 ± 6.3 |
| Insulin | 14.3 ± 7.9 |
| Glucose | 98.5 ± 11.7 |
| Framingham risk score (FRS) | 8.2 (4.9) |
| HOMA-IR | 3.2 (2.8) |
